# Profound thrombocytopenia associated with administration of multiple anti‐inflammatory agents in baboons

**DOI:** 10.1002/iid3.588

**Published:** 2022-01-20

**Authors:** Mohamed H. Bikhet, Christophe Hansen‐Estruch, Mariyam Javed, Dalis E. Collins, Jeremy B. Foote, David Ayares, Hidetaka Hara, David K. C. Cooper

**Affiliations:** ^1^ Xenotransplantation Program, Department of Surgery University of Alabama at Birmingham Birmingham Alabama USA; ^2^ Animal Resources Program University of at Birmingham Birmingham Alabama USA; ^3^ Department of Microbiology University of Alabama at Birmingham Birmingham Alabama USA; ^4^ Revivicor Blacksburg Virginia USA

**Keywords:** baboon, IL‐6, pig, thrombocytopenia, xenotransplantation

## Abstract

Congestion, granular platelet debris both within macrophage and extracellularly, and neutrophil infiltration in the spleen of a baboon that was euthanized with profound thrombocytopenia.

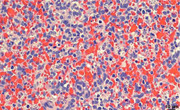

Letter to the Editor

Agents that inhibit the action of IL‐6, either through blockade of IL‐6 receptors (e.g., tocilizumab) or through binding to soluble IL‐6 (e.g., siltuximab), might suppress the systemic inflammatory response documented after pig‐to‐nonhuman primate organ xenotransplantation.^1^ Tocilizumab results in blocking IL‐6 receptors on baboon tissues, but *not* on pig tissues; this results in increased levels of circulating IL‐6, potentially detrimental to the pig graft.^2^ Siltuximab also binds to soluble IL‐6 from baboons, but not from pigs. Whether administration of an agent that inhibits IL‐6 activity is beneficial or detrimental in xenotransplantation, therefore, remains uncertain.^2^

However, we hypothesized that the administration of a combination of both agents may prove beneficial in pig‐to‐baboon organ transplantation by (i) reducing the level of soluble IL‐6 circulating in the blood, thus reducing binding to the pig graft, and (ii) reducing the inflammatory effect following binding of IL‐6 to baboon tissues. We thus included both of them in our regimen, that already included TNF blockade with etanercept.

Kidney transplantation from a GTKO.hCD46.hTBM (thrombomodulin) pig (Revivicor) was carried out in two immunosuppressed baboons receiving an anti‐CD40mAb‐based regimen.^3^ All three agents (tocilizumab [10 mg/kg on Days −1, 7, 14], siltuximab [11 mg/kg iv on Day 0], etanercept [1 mg/kg on Day 0, 0.5 mg/kg on Days 3, 7, 10]) were administered.

The two baboons survived for 15 (B5618) and 22 (B14016) days, respectively, both requiring euthanasia for profound thrombocytopenia, with platelet counts as low as 2.2 × 10^3^ and 33.0 × 10^3^, respectively. The first baboon (B5618) developed a large collection of peritoneal fluid (confirmed by ultrasound) for which it was euthanatized. At necropsy, heavily blood‐stained fluid collections in the peritoneal (approximately 400 ml), pleural, and pericardial spaces were found. The evidence was that the baboon had developed a consumptive coagulopathy.

The second baboon (B14016) followed a similar post‐transplant course, and developed a large fluid collection in the abdomen (confirmed by ultrasound). At this time, its clinical condition deteriorated rapidly and it showed features of having sustained a cerebrovascular accident. We suspected the peritoneal fluid would be heavily blood‐stained, but at necropsy it proved to be serous fluid (approximately 400 ml—but not urine), and there was also a collection in the pericardial cavity. The evidence was that, as a result of thrombocytopenia, the baboon had suffered a cerebral hemorrhage, but the cause of the effusions remained uncertain.

Histopathologic assessment of the pig kidney grafts revealed no evidence of immune‐mediated rejection in B5618, and in B14016 there were occasional fibrin thrombi in small caliber renal interstitial arteries and the surrounding renal interstitium was mildly edematous. Histopathologic assessment of necropsied native organs (heart, spleen, and liver) from B14016 revealed the presence of interstitial hemorrhage and myocardial degeneration in the wall of the left ventricle and interventricular septum of the heart, centrilobular congestion in the liver, and fibrin accumulation admixed with increased numbers of neutrophils in the sinusoids of the red pulp of the spleen (Figure 1), though the spleen was not enlarged.

**Figure 1:**
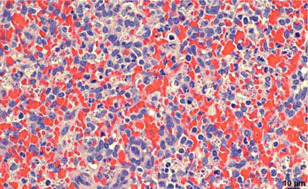
Splenic red pulp from B14016 (H&E, x40). Demonstrates congestion, granular platelet debris both within macrophage and extracellularly, and neutrophil infiltration

IL‐6 increases the number of circulating platelets during inflammation, Furthermore, IL‐6 may act like thrombopoietin and be a regulator of megakaryocytes.^4^, ^5^, ^6^ Tocilizumab is known to cause increased consumption and reduction of platelet count in patients with rheumatoid arthritis through drug‐induced thrombocytopenia and needs to be discontinued if the count falls below 5 × 10^3^. Siltuximab is associated with a reduced incidence of thrombocytopenia.^7^, ^8^, ^9^ Etanercept is another anti‐inflammatory agent known to be associated with isolated thrombocytopenia or aplastic anemia.^10^, ^11^, ^12^

When organs from pigs expressing a human coagulation‐regulatory protein, for example, thrombomodulin, have been transplanted into baboons, we have not seen thrombocytopenia of this degree when tocilizumab and etanercept have been administered together.^13^, ^14^ (In reference^13^, the early development of thrombocytopenia was subsequently found to be associated with a lack of expression of thrombomodulin in the pig kidney graft.) We therefore suggest that the combination of the three agents is potentially detrimental to platelet production and/or maturation, and may induce platelet clearance in the spleen.

A limitation of this study is that we did not measure serum levels of either baboon of pig IL‐6 in these baboons.

## CONFLICT OF INTERESTS

David Ayares is an employee of Revivicor, Blacksburg, VA. The other authors declare that there are no conflict of interests.

## AUTHOR CONTRIBUTIONS

Mohamed H. Bikhet, Christophe Hansen‐Estruch, Mariyam Javed, Dalis E. Collins, Jeremy B. Foote, David Ayares, Hidetaka Hara, and David K. C. Cooper contributed to research, design, analysis. Mohamed H. Bikhet and David K. C. Cooper wrote this manuscript.

## Data Availability

Data sharing not applicable to this article as no datasets were generated or analyzed during the current study.
